# Around the Hospitals

**Published:** 1893-09-02

**Authors:** 


### AROUND THE HOSPITALS.

Notice to the Managers of Institutions.—Special space is now reserved for the insertion of notices of hospital meetings and festivals. The Editor requests that all notices may reach The Hospital Office, 428, Strand, at least one week before the date of each meeting.

**The Hospital for Epilepsy and Paralysis, Regent's Park.**—This hospital affords the gratifying intelligence that it is now out of debt. But this by no means indicates the fact that it has an assured or reliable income sufficient to its wants. On the contrary, had it not been for the assistance of valuable legacies, a most unequal item in the yearly receipts, the general expenses of the hospital could not have been defrayed. The institution has to provide for an expensive class of patients, who by their payments only defray one-third of the expenditure. It is essentially a hospital for progress, and has and is doing valuable work in the special form of disease to which it ministers. At the annual meeting Dr. Althous stated the interesting fact in the history of the hospital and of medicine that here the first case in which the seat of a tumour in the brain was correctly diagnosed, and the growth subsequently removed by surgical operation. Further, he mentioned that important developments in another direction were being carefully tested, which would, if brought to a successful issue, materially aid the treatment of nervous diseases. The number of in-patients averaged about the same as during the last few years, whilst the out-patients in 1892 show an increase of 200. It is noticeable in the table which is given, showing the various occupations of the patients, that male patients are very much in preponderance. Amongst these last those holding posts as clerks are the largest in numbers, followed by carpenters and labourers.

**Guy's Hospital.**—Taking into consideration the locality in which this hospital is situated, and that its wards do not possess the advantages of many more modern buildings, its management is to be congratulated on the low rate of mortality, especially as a very large number of patients received are sufferers from severe accidents. Indeed, the proportion of accidents of all kinds treated in the hospital is very large, and out of the 3,000 surgical cases admitted last year 1,019 were due to accidents. Much attention is paid at Guy's to special branches of medical work, and the maternity work and dental department form an important part of its scheme. An excellent convalescent home in connection with the hospital is resorted to by a large number of patients, to whom it proves most beneficial. Labouring under some disadvantages of structure and locality, Guy's is certainly amongst the pioneer hospitals. Dr. Steele, the late medical superintendent, a man of wide interests and devotion to his work, is much regretted, but both his office and that of the Matron, Miss Victoria Jones, have been ably filled by Dr. Perry and Miss Nott Bower respectively.

